# Structural and Biochemical Analysis of Tyrosine Phosphatase Related to Biofilm Formation A (TpbA) from the Opportunistic Pathogen *Pseudomonas aeruginosa* PAO1

**DOI:** 10.1371/journal.pone.0124330

**Published:** 2015-04-24

**Authors:** Kun Xu, Shanshan Li, Wen Yang, Kan Li, Yuwei Bai, Yueyang Xu, Jin Jin, Yingying Wang, Mark Bartlam

**Affiliations:** 1 State Key Laboratory of Medicinal Chemical Biology, Nankai University, Tianjin, China; 2 College of Life Sciences, Nankai University, Tianjin, China; 3 College of Pharmacy, Nankai University, Tianjin, China; 4 Key Laboratory of Pollution Processes and Environmental Criteria (Ministry of Education), College of Environmental Science and Engineering, Nankai University, Tianjin, China; Centre National de la Recherche Scientifique, Aix-Marseille Université, FRANCE

## Abstract

Biofilms are important for cell communication and growth in most bacteria, and are responsible for a number of human clinical infections and diseases. TpbA (PA3885) is a dual specific tyrosine phosphatase (DUSP) that negatively regulates biofilm formation in the opportunistic pathogen *Pseudomonas aeruginosa * PAO1 by converting extracellular quorum sensing signals into internal gene cascade reactions that result in reduced biofilm formation. We have determined the three-dimensional crystal structure of wild-type TpbA from *P*. *aeruginosa * PAO1 in the phosphate-bound state and a TpbA (C132S) mutant with phosphotyrosine. Comparison between the phosphate-bound structure and the previously reported ligand-free TpbA structure reveals the extent of conformational changes that occur upon substrate binding. The largest changes occur in the functional loops that define the substrate binding site, including the PTP, general acid and α4-α5 loops. We further show that TpbA efficiently catalyzes the hydrolysis of two phosphotyrosine peptides derived from the periplasmic domain of TpbB (YfiN, PA1120), with a strong preference for dephosphorylating Tyr48 over Tyr62. This work adds to the small repertoire of DUSP structures in both the ligand-free and ligand-bound states, and provides a starting point for further study of the role of TpbA in biofilm formation.

## Introduction

*Pseudomonas aeruginosa*, a bacterium noted for its metabolic variety, is found in a wide range of biotic and abiotic habitats including water, soil and various organisms. The versatility of this organism is understood to be related to the large number of regulatory proteins in its genome (469 of 5,570 open reading frames) [1]. As an important opportunistic pathogen, it can cause numerous acute and chronic infections in humans [2] and is now believed to be the third-leading cause of nosocomial infections. Patients with cystic fibrosis and immune-compromised patients are at particularly high risk from *P*. *aeruginosa* infection, and it is considered to be the leading cause of morbidity and mortality among cystic fibrosis (CF) patients [3, 4]. The mucoid phenotype of *P*. *aeruginosa* provides it with an advantage in resisting phagocytosis [5]. In addition, due to the membrane-permeability barrier, *P*. *aeruginosa* has acquired a high level of drug resistance [6], which makes treating patients infected with this pathogen extremely challenging. Critical traits that contribute towards the pathogenicity of *P*. *aeruginosa* include the production of incalculable virulence factors, formation of biofilms and antibiotic resistance [7]. As a result of its considerable morbidity and mortality, *P*. *aeruginosa* has raised considerable concern at both the basic research and clinical levels, and increasing efforts have been made to explore more efficient treatments for *P*. *aeruginosa* infection.

In *P*. *aeruginosa*, biofilm formation is the crucial step in persistence in the host and establishment of chronic infections, and is also responsible for cell growth and communication [8, 9]. For this reason, it is commonly used as a model organism in the study of biofilm formation [10]. As a mechanism of communication to coordinate behavior, quorum sensing has been found to have an influence on biofilm formation [11, 12] and requires a large network of genes for regulation [13–15]. TpbA (PA3885) from *P*. *aeruginosa* PA14 has been reported to be secreted to the periplasm and to link the extracellular quorum-sensing signals to EPS (extracellular polysaccharide) production and biofilm formation via negative regulation of c-di-GMP (3',5'-cyclic diguanylic acid) [16], which regulates biological processes including motility and biofilm formation in bacteria [17]. Furthermore, extracellular DNA (eDNA), which forms a major part of the biofilm matrix, is inversely proportional to c-di-GMP concentrations, and TpbA has been shown to act as a positive regulator of eDNA and cell lysis by reducing c-di-GMP concentrations [18].

In recently published work, it was demonstrated that the Las system indirectly controls the levels of c-di-GMP through tyrosine phosphatase TpbA [16]. TpbA is a dual specific tyrosine phosphatase (DUSP) that has a conserved tyrosine phosphatase domain, and relevant phosphatase activities with p-nitrophenyl phosphate (pNPP) and phosphotyrosine peptides have been reported [16]. To date, TpbB is the only known substrate of TpbA as shown *in vitro* using purified enzymes. TpbA is known to control phosphorylation of TpbB at both Tyr and Ser/Thr residues *in vivo* as detected by mutagenesis [16] and by Western blot analysis [19]. TpbB (PA1120), also known as YfiN, is a multi-domain membrane enzyme connecting periplasmic stimuli to cytosolic c-di-GMP production by an allosteric inside-out signaling mechanism. Thus, TpbA controls the rugose morphology in *P*. *aeruginosa* by dephosphorylating TpbB [19]. This will further influence a number of properties of *P*. *aeruginosa* and eventually suppress biofilm production, as well as enhance swarming motility [16]. Loss or mutation of TpbA can decrease swimming, abolish swarming and increase aggregation, indicating that TpbA is a negative regulator of *P*. *aeruginosa* biofilm formation. Therefore, TpbA may function as a balancing factor between biofilm formation and motility in *P*. *aeruginosa*.

As an important connection between extracellular quorum-sensing signals and biofilm formation, TpbA may be a potential target for controlling bacterial social behaviour and even drug development [20]. In order to provide further understanding of the structure and function of TpbA, we have determined high-resolution crystal structures of TpbA from *P*. *aeruginosa* PAO1 in the ligand-bound state. This represents the first structure of a bacterial periplasmic DUSP in the closed, ligand-bound state and follows on from the recent solution structure of ligand-free TpbA [21]. Our structures reveal that TpbA adopts a canonical eukaryotic-like DUSP fold with additional unique secondary structure elements. A bound phosphate ion is located in the shallow substrate binding pocket of TpbA, enabling us to study the conformational changes that occur upon phosphate binding from comparison with the previously reported ligand-free TpbA structure. An enzyme-product complex solved from co-crystallization of the TpbA C132S mutant with the substrate pTyr suggests the mechanism of dephosphorylation. Finally, our study shows that TpbA can efficiently dephosphorylate two phosphorylated peptides derived from the periplasmic domain of TpbB, with a stronger preference for Tyr48 rather than Tyr62.

## Materials and Methods

### Expression and purification of TpbA

TpbA was expressed and purified as described previously (Yang et al., 2010). Briefly, the gene encoding TpbA (Gene ID 878776; residues 29–218) was amplified from the genome of *Pseudomonas aeruginosa* PAO1 (kindly gifted by Prof. Lei Wang) and then inserted into the expression vector pGEX-6P-1 (GE Healthcare) using the restriction sites *Bam*HI and *Xho*I and with an N-terminal glutathione-*S*-transferase (GST) tag. The recombinant plasmid was transformed into *Escherichia coli* strain BL21 (DE3) and a single colony was cultured in LB medium at 310K with 50 μg/mL ampicillin, to an OD_600_ of 0.6–0.8, then induced with 0.5 mM IPTG (isopropyl-β-D-thiogalactopyranoside) for 18h at 289 K. The cells were harvested by centrifugation, resuspended in phosphate-buffered saline (1×PBS, pH 7.4) and then lysed by sonication on ice. The cell debris was removed by centrifugation at 18,000g for 40 min at 4°C. The supernatant was loaded on to a GST column (GE Healthcare) equilibrated with 1×PBS, then followed by GST tag removal (PPase) and anion-exchange chromatography on a ResourceQ column (GE Healthcare). The purified protein was finally exchanged into a buffer consisting of 20mM Tris, 100mM NaCl for crystallisation. The purity of PA3885 was estimated to be greater than 95% by SDS-PAGE analysis.

Expression and purification of the TpbA (C132S) mutant protein was performed following the same protocol as for the wild-type protein, with one notable exception. The expression and purification of the mutant was performed in Tris instead of PBS buffer to reduce the influence of phosphate.

### Crystallisation of TpbA

The crystallisation of wild-type TpbA has been reported previously [22]. Briefly, the purified TpbA protein was concentrated to 30 mg/mL in a buffer containing 20 mM Tris, pH 8.0, 150 mM NaCl, 10 mM β-mercaptoethanol. Crystallization was performed by the sitting-drop vapor-diffusion method at 20°C (293K): 1 μL protein solution (30 mg/mL) was mixed with 1 μL well solution and equilibrated over 100 μL well solution. Good quality crystals were grown under the optimized conditions of 1.7 M ammonium phosphate monobasic, 0.1 M Tris, pH 8.1 and 7%(v/v) polyethylene glycol (PEG) 400 as additive

To obtain a complex with pTyr, the purified TpbA (C132S) mutant at 30 mg/mL was co-crystallized with pTyr at a protein:substrate molar ratio of 1:5 by the sitting-drop vapor-diffusion method at 20°C (293K). After crystal screening, crystals were obtained under the optimized conditions of 15%(w/v) PEG 10000, 0.1M Citrate pH5.5, 2%(v/v) Dioxane.

### Data collection and processing

Prior to data collection, crystals were cryoprotected by adding 20% glycerol into the crystallization buffer before being flash-cooled in liquid nitrogen. Diffraction data for the wild-type protein were collected on beamline 5A of the Photon Factory, Japan at -173°C (100K). Diffraction data for the TpbA (C132S)-pTyr structure was collected on beamline 17U of the Shanghai Synchrotron Radiation Facility (SSRF). All data were integrated, scaled and merged using the HKL2000 suite of programs [23].

### Structure determination

Attempts to determine the three-dimensional structure of TpbA by molecular replacement proved unsuccessful due to the low sequence identity between TpbA and other DUSPs or protein tyrosine phosphatases. The structure could also not be determined by molecular replacement using the solution structure of *P*. *aeruginosa* TpbA (PDB ID: 2M3V) as a search model. The structure was subsequently phased by molecular replacement using MrBUMP with a full search of the Protein Data Bank [24]. The search identified the structure of a novel phosphatase from *Arabidopsis thaliana* (PDB ID: 1XRI, sequence identity: 23%) as a search model, with final R_work_/R_free_ of 47.2%/50.9% and a Q factor of 53.0%. The molecular replacement solution was sufficient to kick-start tracing of the TpbA model using the AutoBuild module in PHENIX [25]. After an initial round of model building, a total of 310 out of 380 residues were traced in two molecules with R_work_/R_free_ of 33.2%/39.4%, and building of the chain was completed manually in Coot [26]. Refinement was performed in PHENIX with cycles of manual rebuilding in Coot. In the final stage of refinement, the TbpA structure was optimised using the PDB_REDO server [27].

The TpbA (C132S)-pTyr structure was solved by molecular replacement in PHENIX using a single monomer of the wild-type TpbA as a search model. Refinement was performed in PHENIX [25] with cycles of manual rebuilding in Coot [26]. In the final stage of refinement, the structure was optimized using the PDB_REDO server [27]. Structural data are available in the Protein Data Bank under the accession numbers 4R0S for wild-type TpbA and 4R0T for the C132S-pTyr structure.

### Phosphotyrosine peptide dephosphorylation assay

All phosphorylated peptides were synthesized by Scilight Peptide (Beijing, China). To test the tyrosine phosphatase activity of TpbA, an assay was performed using the Tyrosine Phosphatase Assay System (Promega). 20 μg of purified TpbA was incubated with either phosphotyrosine peptide TpbB-1 (LRA(pY)ADPNQ) or peptide TpbB-2 (SIS(pY)TVEAA) in a reaction buffer (50 mM Tris-Acetate, 10 mM MgCl2, pH 5.5) at 37°C for 3 h. The final concentration of substrates used in the experiment were 0, 50, 100, 200, 400, 600, 800 μM. The reaction was quenched using a molybdate dye solution and incubated for 30 min at room temperature. Released phosphate was quantified by measuring the absorbance at 630 nm.

## Results and Discussion

### Structural overview of *P*. *aeruginosa* TpbA

The gene encoding TpbA from residues 29–218 was amplified from the *P*. *aeruginosa* PAO1 genome (kindly gifted by Prof. Lei Wang). The N-terminal 28 amino acids encode a signal peptide necessary for secretion and were therefore not included for structural analysis. Native TpbA crystallises with two molecules in an asymmetric unit (a.s.u.), although our results and those of Koveal and colleagues show that it exists as a monomer in solution [21]. The two molecules in an a.s.u. are continuously visible in the electron density map from residues 34–195 and have an identical conformation, yielding an r.m.s.d. of 0.1 Å for 161 aligned Cα atoms. Data collection and refinement statistics for TpbA are summarised in Table 1.

**Table 1:** Data collection and refinement statistics.

	TpbA	TpbA (C132S)-pTyr
**Data collection statistics**		
**Space group**	*I 2*_*1*_ *2*_*1*_ *2*_*1*_	*I 2*_*1*_ *2*_*1*_ *2*_*1*_
**Unit cell parameters**	a = 80.1, b = 82.8, c = 119.9	a = 78.8, b = 82.6, c = 120.8
**Wavelength (Å)**	1.0000	0.9793
**Resolution (Å)**	50.0–2.03 (2.12–2.03)^a^	50.0–2.60 (2.64–2.60)
***R*_merge_ (%)** ^**b**^	7.2 (67.2)	7.8 (48.0)
***I*/σ(*I*)**	20.2 (2.3)	24.9 (3.4)
**Completeness (%)**	99.1 (99.6)	94.7 (99.8)
**No. of observations**	167,718	46,678
**No. of unique observations**	25,665 (2,549)	11,739 (597)
**Redundancy**	6.5 (5.9)	4.1 (4.0)
**Refinement statistics**		
**Resolution (Å)**	50.0–2.03	50.0–2.60
***R*_work_ / *R*_free_ (%)** ^**c**^	22.8 / 25.0	21.7 / 26.5
**R.m.s. deviations**		
** Bond lengths (Å)**	0.009	0.004
** Bond angles (°)**	1.349	0.739
**MolProbity** ^**d**^		
** Favoured (%)**	96.9	94.9
** Outliers (%)**	0	0
** Rotamer outliers (%)**	0.4	0.4
** Overall score**	1.19	1.74

^a^ Numbers in parentheses are corresponding values for the highest resolution shell.

^b^ *R*_*merge*_ = Σ_h_Σ_l_ | I_ih_-<I_h_> |/Σ_h_Σ_I_ <I_h_>, where <I_h_> is the mean of the observations I_ih_ of reflection h.

^c^ *R*_*work*_ = Σ (||F_p_(obs)|-|F_p_(calc)||)/ Σ|F_p_(obs)|; *R*_*free*_ = R factor for a selected subset (5%) of the reflections that was not included in prior refinement calculations.

^d^ Ramachandran plot calculated using MolProbity.

**Fig 1:**
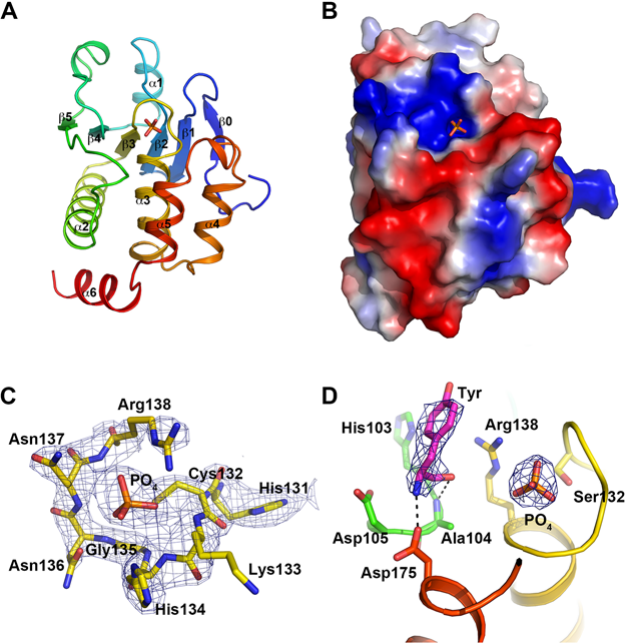
Crystal structure of *P*. *aeruginosa* TpbA. A: Cartoon representation of TpbA coloured from blue at the N-terminus to red at the C-terminus. Secondary structure elements are labelled and the phosphate ion is shown in stick representation. B: Electrostatic surface representation of TpbA. C: The PTP loop (residues 131–138) and phosphate ion are shown in stick representation. A 2mFo-DFc electron density map is shown contoured at 1.2 σ. D: 2mFo-DFc electron density for the orthophosphate and bound tyrosine from the Tpb (C132S)—pTyr structure. Electron density is shown contoured at 1.2 σ.

TpbA adopts a canonical eukaryotic-like DUSP fold with additional secondary structure elements that distinguish it from other DUSPs, including an N-terminal β-strand β0 that caps and extends the β-sheet, and a C-terminal helix α6 that packs on the outside of the protein (Fig 1A). The active site architecture of the DUSP catalytic domain is defined by four loops: the protein tyrosine phosphatase (PTP) loop containing the conserved catalytic cysteine Cys132; the general acid loop containing a conserved aspartic acid that functions as a catalytic acid/base; a variable insert that varies among DUSPs and is predicted to involve in substrate recognition; and the α4-α5 loop that is equivalent to the Q-loop in protein tyrosine phosphatases (PTPs). Together, the four loops define a shallow active site approximately 6 Å deep that is consistent with the selective ability to dephosphorylate pTyr, pSer and pThr residues (Fig 1B). In each TpbA monomer, additional electron density is observed adjacent to the PTP loop that is consistent with a phosphate ion that was introduced from the ammonium phosphate monobasic in the crystallization conditions (Fig 1C). The phosphate binding is typical of DUSP enzymes, with main chain amide atoms in the active site PTP loop and the guanidine group from Arg138 strongly interacting with the oxygen atoms from the phosphate ion (Fig 1C).

### Crystal structure of the TpbA (C132S) mutant with pTyr

TpbA has been shown to possess phosphatase activities for pNPP and phosphotyrosine peptides, and can dephosphorylate pTyr, pSer and pThr residues [16]. In an attempt to provide a structural basis for the phosphatase mechanism, we co-crystallised a C132S mutant of TpbA with pTyr on the basis that a C132S mutant of TpbA was previously reported to be inactive by Koveal and colleagues. The use of Cys → Ser/Ala substrate-trapping mutants is a common strategy used to determine the structures of PTPs in a complex with their target substrates as they typically maintain binding affinity but completely lose activity [28–31].

The TpbA C132S mutant co-crystallized with pTyr shares the same crystal form as native TpbA with two molecules in the a.s.u. Comparison between the wild-type and C132S-pTyr structures reveal an r.m.s.d. of 0.35 Å for 162 aligned Cα atoms, indicating no major differences between the two structures. Close inspection of the electron density maps for the C132S-pTyr structure revealed phosphate alone was present in the active site. In one of the two monomers, however, clear electron density was visible for tyrosine in the shallow binding pocket approximately 5 Å from the bound phosphate, indicating that pTyr had been dephosphorylated (Fig 1D). The tyrosine is trapped close to the active site, and its position overlaps with that of a glycerol molecule in the wild-type structure. The trapped tyrosine stacks adjacent to His76 and is coordinated by a 3.1 Å hydrogen bond between the carbonyl oxygen and the amide group of Ala104, and a 2.5 Å hydrogen bond between the amide group and the side chain of Asp175. The side chain of Asp105, the proposed general acid/base, is approximately 3.8 Å from the amide group of the trapped tyrosine, and a weak electrostatic interaction (3.6 Å) is provided between the carbonyl oxygen and the Nε atom of Arg138. This observation suggests that Cys → Ser/Ala mutants of TpbA may not be ideal for the isolation of substrates, and further work is underway to explore other more effective substrate-trapping mutants.

The DUSPs, which are a subset of the type 1 cysteine-based PTPs, have a PTP loop with the consensus catalytic sequence HCXXXXXR and feature a conserved aspartic acid residue that is important for catalysis [32]. In TpbA, this catalytic sequence corresponds to ^131^HCKHGNNR^138^, with Cys132 confirmed to function as the catalytic nucleophile during catalysis [21]. The role of general acid/base in DUSPs is fulfilled by an aspartic acid residue in the general acid loop. In TpbA, the general acid loop features three aspartic acid residues, only two of which—Asp105 and Asp108—are conserved among *Pseudomonas* species (S1 Fig). Asp105 was predicted to be the most likely candidate for the general acid/base by Koveal and colleagues on the basis that (a) it can access the active site without the need for large global rearrangements in structure, and (b) a D105A mutant resulted in a 53% decrease in catalytic efficiency. The observation from our pTyr structure that the bound tyrosine in the active site is coordinated by Asp105 lends further support for the role of Asp105 as the general acid/base in the dephosphorylation reaction catalysed by TpbA.

### Comparison with other DUSPs

Protein tyrosine phosphatases (PTPs) and the subset of dual-specificity phosphatases (DUSPs) share a common catalytic mechanism but have very low sequence homology, with the exception of the loops that define the active site [33]. A DALI search for structural similarity to TpbA identified a number of related DUSP structures, although sequence identity was less than 20% in the majority of cases. The closest match is to human protein phosphatase 23 (DUSP23, PDB ID: 2IMG, Z-score 17.2, r.m.s.d. 2.2 Å, sequence identity: 19%), followed by the phosphatase from the *Arabidopsis thaliana* gene locus At1g05000 (PDB ID: 1XRI, Z-score 16.7, r.m.s.d. 2.1 Å, sequence identity: 23%). TpbA is similar to a number of other human DUSPs, including MAP kinase phosphatase 5 (DUSP10, PDB ID: 1ZZW, Z-score 16.3, r.m.s.d. 2.3 Å, sequence identity: 14%), the cell cycle protein Cdc14B (PDB ID: 1OHC, Z-score 16.0, r.m.s.d. 2.8 Å, sequence identity: 15%), DUSP5 (PDB ID: 2G6Z, Z-score 15.8, r.m.s.d. 2.2 Å, sequence identity: 11%), DUSP19 (PDB ID: 3S4E, Z-score 15.8, r.m.s.d. 2.2 Å, sequence identity: 11%), and DUSP4 (PDB ID: 3EZZ, Z-score 15.5, r.m.s.d. 2.3 Å, sequence identity: 13%). The closest similarity to a bacterial DUSP is the putative phosphatase DUF442 from the marine bacterium *Shewanella putrefaciens* CN-32 (PDB ID: 3GXH, Z-score 14.5, r.m.s.d. 2.6 Å, sequence identity 21%). Unsurprisingly, similarity was also observed to the NMR structure of *P*. *aeruginosa* TpbA in the ligand-free state (PDB ID: 2M3V, Z-score 12.1, r.m.s.d. 3.8 Å, sequence identity: 93%).

**Fig 2:**
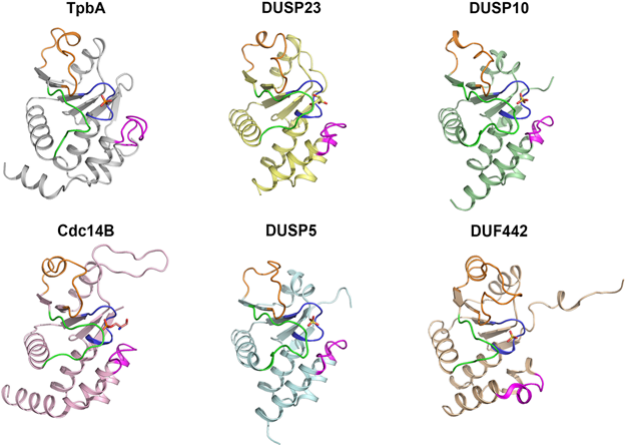
Comparison of TpbA with its closest structural homologs. A: Crystal structures of TpbA (grey), DUSP23 (PDB ID: 2IMG, pale yellow), DUSP10 (PDB ID: 1ZZW, pale green), Cdc14B (PDB ID: 1OHE, light pink), DUSP5 (PDB ID: 2G6Z, pale cyan) and DUF442 (PDB ID: 3GXG, wheat). Functionally important loops are coloured as follows: PTP loop, blue; general acid loop, green; variable insert, yellow; α4-α5 loop, purple.

The human DUSP23 structure, also known as VHZ, has been reported in the ligand-bound state with a malate ion (PDB ID: 2IMG) [34] and with metavanadate (PDB ID: 4ERC) [35]. In the malate-bound structure, a tyrosine residue from a neighbouring symmetry related monomer is located in the shallow binding cleft, mimicking the position of tyrosine in an enzyme-substrate/product complex. However, the presence of malate reportedly presents a catalytically unfavourable conformation of the general acid loop and distorts the active site into a shallow and broad crevice reminiscent of DUSPs [35]. The core TpbA and DUSP23/VHZ structures are broadly similar, but one striking difference is the position of the general acid loop, also known as the IPD loop in DUSP23/VHZ for its analogy to the WPD loop in classical PTPs. The closed ligand-bound structures of DUSP23 have a general acid loop approximately 6 Å from the ligand binding site at its closest point, positioning the general aspartic acid in a suitable position for protonation of the scissile oxygen atom [34, 35]. This conformation of the general acid loop is also conserved in the DUSP10, Cdc14B and DUSP5 structures (Fig 2). In the TpbA structure, however, the general acid loop is situated further away from the ligand binding site and the general acid Asp105 is pointing away from the ligand binding site in a catalytically unfavourable position. One explanation is that this conformation of the general acid loop may represent a product release state, as movement of the general acid loop is known to be linked to substrate binding [36]. Further evidence for this is provided by the C132S-pTyr structure (Fig 1D), which is found to be in a post-catalytic state and shares the same conformation of the general acid loop. A similar conformation is also shared by the phosphate-bound bacterial DUSP DUF442 (Fig 2).

**Fig 3:**
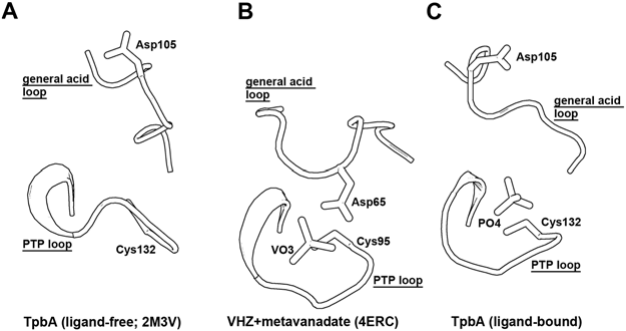
Different states of TpbA. A: The general acid loop and PTP loop of TpbA in the ligand-free state (PDB ID: 2M3V), representing an open state. B: The general acid loop and PTP loop of DUSP23/VHZ in complex with metavanadate (VO3; PDB ID: 4ERC), representing a closed state and showing the catalytically favourable position of the aspartic acid residue. C: The general acid loop and PTP loop of TpbA in the phosphate-bound state, representing a post-catalytic half-open state. The loops are shown in cartoon representation and the catalytic cysteine and aspartic residues are shown in stick representation. All structures are shown in the same orientation after superposition.

The catalytic WPD loop in PTPs, equivalent to the general acid loop in DUSPs, has been captured in four different conformations: an open state, a closed state, an intermediate state and an atypical open state [37]. However, substrate binding alone is not sufficient to induce closure of the WPD loop. A conserved water molecule in PTPs with a closed WPD loop is absent or displaced in structures with an open WPD loop, suggesting that it is a key part of the closure mechanism. No such water is present in either the TpbA or DUF442 structures, and indeed no equivalent water has been observed in DUSP structures to date. The above analysis suggests a mechanism in which the general acid loop folds over the active site upon substrate binding, as in the DUSP23/VHZ structures, bringing the catalytic aspartic acid residue into a favourable position for protonation of the scissile oxygen atom of the substrate (Fig 3). Following the reaction, the general acid loop then adopts a half-open conformation to allow release of the product, as shown in the TpbA and TpbA C132S-pTyr structures (Fig 3). Further structural studies are needed to capture different states of TpbA along the reaction pathway in order to understand the conformation of the general acid loop.

### Comparison of ligand-bound and ligand-free TpbA

Substrate binding by PTPs has been extensively studied and is associated with a rotation of the general acid loop and a large movement of the catalytic acid/base of up to 10 Å, switching the enzyme from an open inactive state to a closed active state [36]. In contrast, relatively few DUSPs have been studied in the open conformation and thus understanding of the changes that occur in DUSPs upon substrate binding and catalysis is more limited. In the first such study of a bacterial periplasmic DUSP, Koveal and colleagues recently reported the solution structure of *P*. *aeruginosa* TpbA in the ligand-free state, and used NMR to study the active site dynamics and conformational changes that occur upon phosphate binding [21]. Our structure of TpbA in the phosphate-bound state provides a unique opportunity to compare the structures of TpbA in two distinct states in order to understand the conformational changes that occur upon phosphate binding.

**Fig 4:**
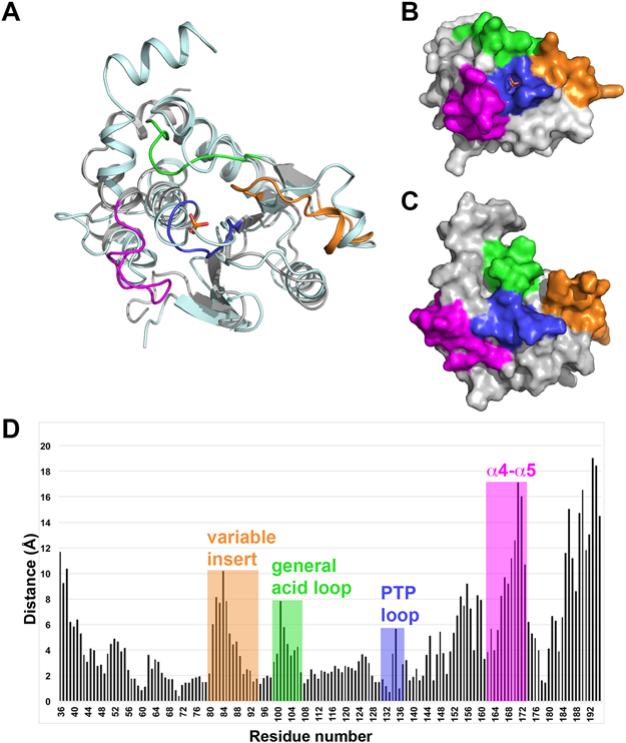
Comparison of TpbA in the ligand-bound and ligand-free state. A: Superposition of TpbA in the ligand-bound and ligand-free states. Ligand-bound TpbA is coloured grey and functionally important loops are coloured as described in Fig 2. Ligand-free TpbA is coloured pale cyan. B and C: Surface representations of TpbA in the ligand-bound and ligand-free (PDB ID: 2M3V) states, respectively. Functionally important loops are coloured as described in Fig 2. D: Histogram showing the distance (Å) between equivalent residues in the ligand-bound and ligand-free structures.

The ligand-bound and the lowest energy ligand-free TpbA structures can be superimposed with an r.m.s.d. of 2.6 Å for 120 aligned residues, indicating significant conformational changes exist between the two structures. The core DUSP fold is essentially unchanged, but the largest structural changes occur in the PTP, general acid and α4-α5 loops, and at the C-terminal of the protein (Fig 4A, 4B and 4C). The PTP loop, which binds phosphate, undergoes a maximum shift in Gly135 of 5.9 Å to accommodate the phosphate ion. The general acid loop, which is disengaged from the active site in the ligand-free state, becomes positioned close to the PTP loop via a 3.1 Å interaction between the backbone carbonyl of Thr102 and the conserved PTP arginine, Arg138. This conserved Arg138 also stabilises the variable insert via a 3.8 Å interaction with the backbone carbonyl of Phe81, and is positioned 3.5 Å from a glycerol molecule derived from the crystallisation conditions. The conserved arginine in the PTP motif is known to make polar contacts with a residue in the variable insert in some DUSPs, though no such interaction exists in the ligand-free state and the variable insert is disengaged from the active site. In the ligand-bound state, Arg138 in the PTP loop makes a 3.1 Å hydrogen bond with the main chain carbonyl group of Thr102 in the general acid loop. It also makes a weak electrostatic interaction (3.7 Å) with the main chain carbonyl of Ile82 in the variable insert. This arginine is conserved in all PTPs, providing stability to the PTP loop, and mutation at this site has been shown to abolish enzyme activity [38]. A similar arrangement is observed in the DUSP23/VHZ structure, with Arg101 in the PTP loop forming a 2.8 Å hydrogen bond with the carbonyl group of Ile63 in the general acid loop, and a weak electrostatic interaction (3.6 Å) with the carbonyl group of Leu42 in the variable insert. Similarly in the Cdc14B structure, Arg320 in the PTP loop forms a 3.1 Å hydrogen bond with the carbonyl group of Phe285 in the general acid loop and a weak electrostatic interaction (3.5 Å) with the carbonyl group of Leu263 in the variable insert.

The α4-α5 loop of TpbA folds away from the PTP loop in the ligand-free state and adopts a similar conformation to the equivalent loop in the bacterial DUSP DUF442 structure (Fig 2). In the phosphate-bound structure of TpbA, the α4-α5 loop adopts a similar arrangement to that observed in other ligand-bound DUSP structures, including DUSP23 and Cdc14B, whereby the loop folds towards the PTP loop and defines the upper boundary of the PTP pocket (Fig 2). This rearrangement from the ligand-free to ligand-bound conformation involves a large movement of the α4-α5 loop, with the greatest shift of more than 15 Å for residue Asp171 (Fig 4D).

**Fig 5:**
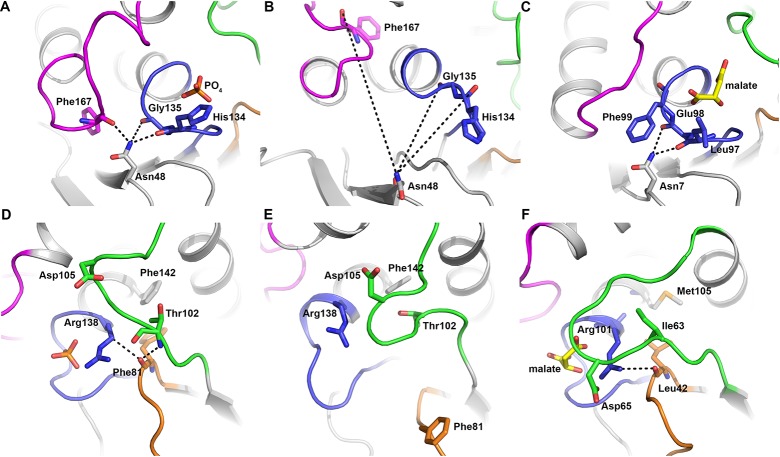
Structural changes in the PTP loop involving. (A) Asn48 in the ligand-bound TpbA structure, (B) Asn48 in the ligand-free structure (PDB ID: 2M3V), and (C) Asn7 in the structure of DUSP23 (PDB ID: 2IMG). Structural comparison of the general acid loop conformation in (D) the ligand-bound TpbA structure, (E) the ligand-free structure (PDB ID: 2M3V) and (F) the DUSP23 structure (PDB ID: 2IMG). Structures are coloured grey and functionally important loops are coloured as described in Fig 2.

Koveal and colleagues highlighted Asn48 as involving in structural changes in the TpbA PTP loop upon ligand binding. In the ligand-bound TpbA structure, Asn48 makes polar contacts with the main chain carbonyls of His134 (3.0 Å) and Gly135 (3.5 Å). It also makes an additional polar contact with the main chain carbonyl group of Phe167 (3.0 Å) in the α4-α5 loop, bringing it into close proximity to the PTP loop (Fig 5A). Asn48 in the ligand-free protein is situated approximately 11 Å from the main chain carbonyl groups of His134 and Gly135 in the PTP loop (Fig 5B). In the malate-bound DUSP23/VHZ structure, the side-chain of Phe99 in the PTP loop is inserted between Asn7 and the α4-α5 loop, such that the loop is positioned approximately 7 Å from Asn7 (Fig 5C).

The variable insert, which Koveal and colleagues reported was unaffected by phosphate binding, also undergoes a substantial conformational change as it is directed from the outside of the ligand-free structure inwards towards the core of the phosphate-bound protein. Ser80, the only residue in the variable insert found to show a chemical shift perturbation upon phosphate binding by Koveal and colleagues, is shifted by 5.5 Å from the ligand-free to the ligand-bound structure. The variable insert changes conformation such that Phe81, facing outwards on the surface of the ligand-free protein, moves by 7.5 Å in the phosphate-bound structure and is directed inwards to stack against Phe142 (Fig 5D and 5E). This π-stacking arrangement appears to be unique to TbpA as similar DUSPs feature valine or leucine in the equivalent position to Phe81, although the conformation of the variable insert in the local vicinity of the binding pocket is similar between ligand-bound DUSPs (Fig 5F).

In comparison to other DUSPs, TpbA features an additional helix α6 which, in the ligand-free state, does not form any stable contacts with the rest of the phosphatase domain. In phosphate-bound TpbA, helix α6 packs on the outside of the phosphatase domain oblique to helix α2, where it is stabilised by polar contacts between Arg117 on helix α2 and the backbone carbonyl of Ala191 and Gly195 on α6. Interestingly, structural comparisons show that helices α5 and α6 of TpbA adopt a similar conformation to α6B of Cdc14B, which features a kink at Lys363 (Fig 2). The functional role of helix α6 remains to be established, but it has been shown to be required for solubility of TpbA as deletion of the helix results in insoluble expression [21].

### Dephosphorylation of periplasmic tyrosines in TpbB

**Fig 6:**
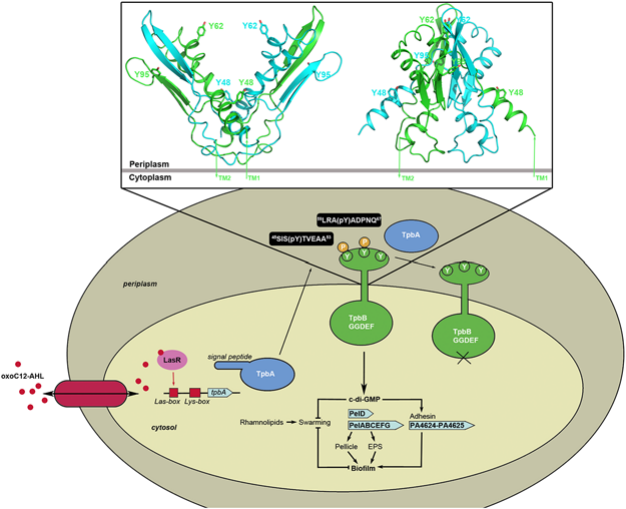
Schematic showing the regulation of biofilm formation in *P*. *aeruginosa* by TpbA (adapted from Ueda and Wood, 2009). Inset is an atomic model of the periplasmic LapD-like domain of TpbB showing the putative locations of the three tyrosine residues: Tyr48, Tyr62 and Tyr95. The LapD-like domain of TpbB is predicted to form a domain-swapped dimer, with the monomers coloured green and cyan. The model of the TpbB LapD-like domain was generated by Swiss-Model (http://swissmodel.expasy.org) using the *Pseudomonas fluorescens* LapD structure (PDB ID: 3PJV) as a template.

TpbA is known to negatively regulate biofilm formation in *P*. *aeruginosa* via dephosphorylation of the membrane-anchored GGDEF protein TpbB (PA1120), also known as YfiN, in the periplasm [16]. It also negatively controls cellular c-di-GMP concentrations, which is believed to occur via TpbB. In their model for regulation of biofilm formation by TpbA, Ueda and Wood proposed that *tpbA* expression is activated by the quorum sensing molecule *N-*(3-oxododecanoyl)-L-homoserine lactone (3-oxoC12-HSL) binding to the LasR transcription factor (Fig 6). TpbA containing the N-terminal signal peptide is translocated into the periplasm where it dephosphorylates TpbB at a tyrosine residue, thus deactivating GGDEF protein activity. As a result, reduced cellular c-di-GMP concentration decreases expression of the *pel* operon and adhesin genes, leading to reduced extracellular polysaccharide (EPS) production, biofilm formation, pellicle formation and enhanced swarming motility (Fig 6).

TpbB has three periplasmic tyrosines (Tyr48, Tyr62 and Tyr95) that could potentially be dephosphorylated by TpbA. Ueda and Wood previously showed that Tyr48 and Tyr62 are required for TpbB activity as both Y48F and Y62F mutants resulted in reduced aggregation. Tyr95, on the other hand, was found not to be phosphorylated/dephosphorylated as a Y95F mutant did not reduce aggregation. Although TpbB is the only known substrate of TpbA to date, the amino acid sequence that is recognised by TpbA is currently unknown. Phosphotyrosine peptides are often used to indicate that a protein possesses tyrosine phosphatase activity. Therefore, to test whether TpbA does indeed target specific TpbB substrates and to obtain more detailed kinetic analysis of TpbA towards its substrates, we synthesized phosphorylated peptides derived from the periplasmic region of TpbB containing Tyr48 (peptide TpbB-1, sequence ^45^SIS(pY)TVEAA^53^) or Tyr62 (peptide TpbB-2, sequence ^59^LRA(pY)ADPNQ^67^). We did not include a peptide containing Tyr95 in our studies as Ueda and Wood have previously shown that Tyr95 of TpbB is not dephosphorylated by TpbA [16].

Tyrosine phosphatase assays confirmed that TpbA efficiently hydrolyses both TpbB peptides 1 and 2, and that activity towards peptide TpbB-1 harbouring Tyr48,is markedly higher than activity towards peptide TpbB-2 harbouring Tyr62. Table 2 shows the *k*_cat_, *K*_M_ and *k*_cat_/*K*_M_ values for the two peptide substrates. Our kinetic analysis indicates that the catalytic activity of TpbA against TpbB phosphotyrosine peptide substrates, which have different *K*_M_ values but similar *k*_cat_ values, increases ~20-fold over the activity for pNPP (*k*_cat_ = 0.0076 s^-1^) (Table 2). This value is consistent with the previously reported *k*_cat_ value for TpbA of 0.00162 s^-1^ with pNPP [21], and *k*_cat_ value of 0.009 s^-1^ with pNPP for DUSP23/VHZ [35, 39]. Our kinetic analysis lends further support to the hypothesis by Ueda and Wood that TpbB is the most likely target protein for TpbA, and that Tyr48 and Tyr62 in the periplasmic domain of TpbB are targets for tyrosine phosphorylation [16]. Furthermore, our results are consistent with the observation that a TpbB Y48F mutant decreased aggregation for 43% of cultures, whereas a Y62F mutant only decreased aggregation for 24% of cultures [16], further indicating that both tyrosines are targets for phosphorylation/dephosphorylation of TpbB with Tyr48 being the strongly preferred site.

**Table 2:** Kinetic constants for the hydrolysis of two periplasmic TpbB peptide substrates by TpbA.

Substrate	*k*_cat_ (s^-1^)	*K*_M_ (μM)	*k*_cat_/*K*_M_ (x10^3^ M^-1^ s^-1^)	Fold difference in *k*_cat_/*K*_M_
^**45**^ **LRA(pY)ADPNQ** ^**53**^	0.149 ± 0.022	49.6 ± 2.1	3.00	1.00
^**59**^ **SIS(pY)TVEAA** ^**67**^	0.167 ± 0.032	111.2 ± 5.4	1.50	0.50
**pNPP**	0.0076 ± 0.0005	1,620 ± 210	0.00469	0.00156

Kinetic constants for the hydrolysis of pNPP are included for comparison.

To date, the allosteric signaling mechanism of TpbB connecting periplasmic stimuli to cytosolic c-di-GMP production remains unclear. Recently, Giardini and colleagues reported the crystal structure of the catalytic GGDEF domain of TpbB, and used a homology model for the full-length protein to propose a model for allosteric regulation of TpbB [40]. The periplasmic PAS domain of TpbB assumes a domain-swapped LapD-like fold and is proposed to function as the driving force for TpbB dimerization. A clearer indication of how TpbA acts on the PAS domain of TpbB was gained from a homology model for the periplasmic LapD-like domain of TpbB, constructed using the crystal structure of the periplasmic output domain of *Pseudomonas fluorescens* LapD (PDB ID: 3PJV) as a template [41]. The PAS domain model is a V-shaped domain-swapped dimer (Fig 6), with each arm of the fold consisting of two α-helices and two β-strands of one monomer and complemented by two β-strands flanked by helical segments from the other monomer. A model of the TpbB LapD-like domain enables us to map the approximate positions of the tyrosine residues predicted to be dephosphorylated by TpbA. The region of TpbB from residues 44–72, which includes both tyrosines that are targeted by TpbA, forms a highly conserved helix α1 with sequence aLrx**Y**axxNlxLiaRsxx**Y**TxEaavvFxD that is highly exposed (Giardina et al., 2013). Tyr48 is exposed on the N-terminal of α -helix 1 that flanks the LapD-like domain, while Tyr62 is located at the top of the V on the inner surface of the arm (Fig 6). Both are surface exposed and should be accessible to TpbA for dephosphorylation, with Tyr48 being in a more favourable position. The location of Tyr62 on the inner surface of the V may place greater restrictions on TpbA and this could account for the lower activity at this site. Tyr95, which was found not to be dephosphorylated by TpbA [16], is located on the underside of the β-sheet and is presumably inaccessible to TpbA (Fig 6). Dephosphorylation of TpbB by TpbA has a crucial role in signalling across the membrane to turn off the GGDEF protein activity and therefore reduce c-di-GMP levels, and further work is underway to study the mechanism of this TpbA-induced signalling using structural and functional analysis.

### Conclusions

We have determined the first ligand-bound crystal structure of *Pseudomonas aeruginosa* TpbA, a bacterial periplasmic DUSP. Comparison with the recent solution structure of TpbA in the open ligand-free state reveals the extent of the conformational changes that occur upon ligand binding. This adds to the small repertoire of DUSP structures that have been determined in both the ligand-free and ligand-bound states, and should provide greater understanding of the relationship between protein dynamics and phosphatase activity and substrate selectivity.

The role of tyrosine phosphorylation and biofilm formation in *P*. *aeruginosa* is still not widely understood. TpbA has been shown to act as a negative regulator of cellular c-di-GMP formation, and loss of TpbA leads to increased concentrations of c-di-GMP that result in enhancement of biofilm formation and inhibition of motility. TpbB has been suggested to be a substrate of TpbA, and we have demonstrated that TpbA has efficient catalytic activity against two phosphorylated peptides derived from the periplasmic domain of TpbB. Our experiments show that catalytic activity of TpbA against the phosphorylated TpbB peptides is ~20-fold higher than against the nonspecific substrate pNPP, which is in line with the ~50-fold increase in activity for VHZ against phosphorylated peptides over the activity against pNPP [35]. This should provide a starting point for further study of the role of TpbA in biofilm formation via regulation of the GGDEF activity of TpbB, and to explore the potential of TpbA for drug development.

## Supporting Information

S1 Fig Multiple sequence alignment of *P*. *aeruginosa* TpbA. Key: P. aeruginosa, *Pseudomonas aeruginosa* PAO1; P. fluorescens, *Pseudomonas fluorescens* (sequence identity 52%); P. mendocina, *Pseudomonas mendocina* (sequence identity 49%); Geobacter, *Geobacter metallireducens* (sequence identity 31%); Acinetobacter, *Acinetobacter baumannii* (sequence identity 27%); Desulfococcus, *Desulfococcus oleovorans* (sequence identity 31%); Burkholderia, *Burkholderia cepacia* (sequence identity 40%); LKHP9428, *Homo sapiens* LKHP9428 (sequence identity 47%). Sequence alignment was generated by CLUSTAL O and visualized by ESPript. Secondary structure elements are shown for *P*. *aeruginosa* TpbA above the alignment.
(PDF)
