# Supplementary material for: Structural and Biochemical Analysis of Tyrosine Phosphatase Related to Biofilm Formation A (TpbA) from the Opportunistic Pathogen Pseudomonas aeruginosa PAO1
Source: PLoS One. 2015 Apr 24;10(4):e0124330. doi: 10.1371/journal.pone.0124330 (PMC4409338; doi:10.1371/journal.pone.0124330)
Supplement: S1 Fig — Key: P. aeruginosa, Pseudomonas aeruginosa PAO1; P. fluorescens, Pseudomonas fluorescens (sequence identity 52%); P. mendocina, Pseudomonas mendocina (sequence identity 49%); Geobacter, Geobacter metallireducens (sequence identity 31%); Acinetobacter, Acinetobacter baumannii (sequence identity 27%); Desulfococcus, Desulfococcus oleovorans (sequence identity 31%); Burkholderia, Burkholderia cepacia (sequence identity 40%); LKHP9428, Homo sapiens LKHP9428 (sequence identity 47%). Sequence alignment was generated by CLUSTAL O and visualized by ESPript. Secondary structure elements are shown for P. aeruginosa TpbA above the alignment. (PDF) [file pone.0124330.s001.pdf]

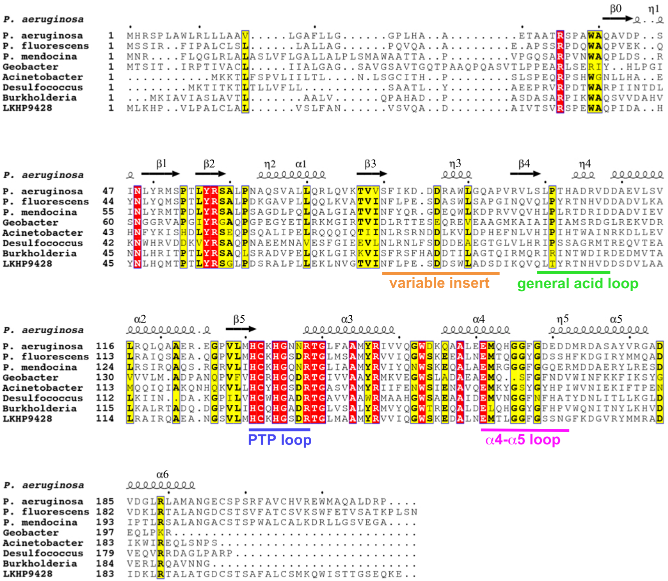
P. aeruginosa
β0 η1
P. aeruginosa 1 MHRSPLAWLRLLLAAV.....LGAFLLG......GPLHA..A.........ETAATRSPAWAQAVDP..S
P. fluorescens 1 MSSIR..FIPALCLSL.....LALLAG......PQVQA..A........E.APSSRPDQWAQPVEK..Q
P. mendocina 1 MNR....FLQGLRLALASLVFLGALLALPLSMAWAATTA..P........VPGQSARPVNWAQPLDS..R
Geobacter 1 MTSIT..IRPTIVACL.....IILGAG..SAVGSAVTGQTPAAQPQASVTLPPEARLSERIY..HLPGI
Acinetobacter 1 ...........MKKTLFSPVLIILTL....N.LSGCITH..P........SLSPEQRPSHWGNLLHA..E
Desulfococcus 1 ........MKTITKTLTLLVFLL.........SAATL..Y.........AEEPRVRPDTWARPIINTDL
Burkholderia 1 ...MKIAVIASLAVTL.......AALV......QPAHAD..P........ASDASARPIKWAQSVVDAHV
LKHP9428 1 MLKHP..VLPALCLAL......VSLFAN......VSVQA..D........AIVTSVRSPEWAQPIDA..H
P. aeruginosa
β1 β2 η2 α1 β3 η3 β4 η4
P. aeruginosa 47 INLYRMSPTLYRSALPNAQSVALLQRLQVKTVVSFIKD.DRAWLGQAPVRVLSLPTHADRVDDAEVLSV
P. fluorescens 44 YNLYQMSPTLYRSALPDKGAVPLLQQLKVATVINFLPE.SDASWLSAPGINQVQLPYRTNHVDDADVLKA
P. mendocina 55 INLYRMTPDLYRSALPSAGDLPQLQALGIATVINFYQR.GDEQWLKDPRVVQVHLPLRTDRIDDADVIEV
Geobacter 60 SNGGRVAPGIYRGAQPGPEGYETLRKMGIRTVIDLRTTESEQREVEAAGMKAIAIPIAMSRDGLREKVDR
Acinetobacter 43 HNFYKISHDLYRSEQPSQALIPELRQQQIQTIINLRSRNDDLKVLDPHEFNLVHIPIHTWAINRKDLLEV
Desulfococcus 42 KNWHRVDDKVYRSAQPNAEEMNAVESFGIEEVLNLRNLFSDDDEAEGTGLVLHRIPSSAGRMTREQVTEA
Burkholderia 45 NNLHRITPTLYRSAQLSRADVPELQKLGIRKVISFRSFHADDTILAGTQIRMQRIRINTWDIRDEDMVTA
LKHP9428 45 YNLHQMTPTLYRSGLPDSRALPLLEKLNVGTVINFLPE.SDDSWLADSDIKQVQLTYRTNHVDDSDVLAA
variable insert
general acid loop
P. aeruginosa
α2 β5 α3 α4 η5 α5
P. aeruginosa 116 LRQLQAAER.EGPVLMHCKHGNNRTGLFAAMYRIVVQGWDKQAALEEMQHGGFGDEDDMRDASAYVRGAD
P. fluorescens 113 LRAIQSAEA.QGPVLMHCKHGSDRTGLMSAMYRVVIQGWSKEEALNEMTQGGYGDSSHFKDGIRYMMQAD
P. mendocina 124 LRSIRQAQS.RGRVLIHCKHGQNRTGLIAAMYRVIYQNWSKEQALAEMRGGFGGQERMDDAERYLRESD
Geobacter 130 VVVLM.ADPANQPVFVHCRHGQDRTGIVVAAYRMKVEGWSLADAEAEMQ..SFGFNDVWINFKKFIKSYG
Acinetobacter 113 MQQIQIAKQNHQKVLLHCYHGSDRTGASVAMYRIIFENWSIENAVQEMKYGSYGYHPIWVNIEKIFTPEN
Desulfococcus 112 LKIIN..DA.KGPILVHCWHGADRTGAVVAAWRMAAHGWSAEAAIDEMVNGGFNFHATYDNLITLLKGLD
Burkholderia 115 LKALRTADQ.DGPVLIHCQHGADRTGLVSALYRMVYQGWTREQALDELQHGGYGFHPVWQNITNYLKHVD
LKHP9428 114 LRAIRQAEA.NGSVLMHCKHGSDRTGLMAAMYRVVIQGWSKEDALNEMTLGGFGSSNGFKDGVRYMMRAD
PTP loop
α4-α5 loop
P. aeruginosa
α6
P. aeruginosa 185 VDGLRLAMANGECSPSRFAVCHVREWMAQALDRP....
P. fluorescens 182 VDKLRTALANGDCSTSVFATCSVKSWFETVSATKPLSN
P. mendocina 193 IPTLRSALANGACSTSPWALCALKDRLLGSVEGA....
Geobacter 197 EQLPKR................................
Acinetobacter 183 IKWIREQLSNPS..........................
Desulfococcus 179 VEQVRRDAGLPARP........................
Burkholderia 184 VERLRQAVNNG...........................
LKHP9428 183 IDKLRTALATGDCSTSAFALCSMKQWISTTGSEQKE..
